# Undertreated Breast Cancer in the Elderly

**DOI:** 10.1155/2013/893104

**Published:** 2013-01-10

**Authors:** Manmeet Kaur Malik, Paul Ian Tartter, Rachel Belfer

**Affiliations:** Division of Breast Surgery, Department of Surgery, St. Luke's-Roosevelt Hospital Center, New York, NY, USA

## Abstract

The effect of undertreatment with adjuvant hormonal therapy, chemotherapy, or radiation was studied in elderly women with breast cancer. A prospectively maintained database was used to identify women undergoing potentially curative surgery between 1978 and 2012. The presentation, pathologic findings, treatment, and outcomes of 382 women over 70 were compared to the findings in 2065 younger patients. Subsequently, conventionally treated and undertreated elderly patients were identified and their characteristics and outcomes were compared. Both young and old patients presented most frequently with mammographic findings, but older patients presented more frequently with mammographic masses while younger patients presented more frequently with mammographic calcifications. Cancers of older patients were significantly more favorable than cancers in younger patients: smaller, with more infiltrating lobular, fewer ductal carcinoma in situ, and more frequently estrogen receptor positive and fewer were poorly differentiated. Elderly patients had less axillary sampling, fewer mastectomies, less adjuvant radiation therapy, and more hormonal therapy. Fifty-one percent of the 382 elderly patients were undertreated by conventional criteria. Undertreated patients were more frequently in situ, better differentiated, smaller, and more often estrogen receptor positive. Forty-four percent of the undertreated patients died during followup without disease recurrence. Despite undertreatment, local and distant disease-free survival was comparable to patients who were not undertreated.

## 1. Introduction

The population of elderly individuals in the United States is increasing. Between 2000 and 2010 the population of women aged 65 and over increased by 11.3% with those 70 and over increasing by 6.4% [1]. According to the Surveillance Epidemiology and End Results (SEERs) database, from 2000 to 2009 the median age for breast cancer diagnoses in the USA was 61 years of age. Approximately 41% were diagnosed above the age of 65, of which 21% were above the age of 75 [2]. As the USA population of women over 65 increases, breast cancer in older individuals has and will continue to become more prevalent. 

The management of breast cancer in the elderly has been a topic of debate. There is a lack of evidence on the optimal management of this group of patients secondary to low enrollment in randomized clinical trials [3, 4]. As a result, treatment decisions have been largely based on studies in younger patients which may not be applicable to elderly patients with breast cancer. Breast cancers in elderly women compared to younger women are histologically less aggressive and have a good response to hormonal therapy. This favorable biologic profile impacts the decision as to whether an elderly patient should be subjected to adjuvant therapy. 

The consequences of these considerations are that elderly patients are often undertreated when compared to younger patients [5–7], but the question that needs to be answered is are there any clinical ramifications to the undertreatment of breast cancer in elderly women [6, 8, 9]? Diab and colleagues demonstrated that the impact of breast cancer on the expected survival of these elderly patients decreases with age [9] and the risk of dying from comorbid conditions often exceeds the risk of cancer recurrence and breast cancer mortality [10]. Although recommendations based on expert opinion are emerging, there is a paucity of level 1 evidence [11]. Determining the optimal treatment for an elderly patient depends largely on clinical judgement, weighing the patients' comorbid conditions with the biology of the tumor. 

## 2. Methods

The senior author (P. I. Tartter) has created and maintained a breast cancer database with the followup of patients who have been cared for by him at Mount Sinai Hospital (1977–1999) or at St. Luke's-Roosevelt Hospital Center (1999–2012). Women 71 years of age and older at the time of diagnosis (*n* = 382) were identified and compared to women younger than 71 years of age at the time of diagnosis (*n* = 2065). We picked the age of 71 as a cutoff to facilitate comparison to other studies. 

Data was collected on age, clinical presentation, mammographic findings, diagnostic method, histopathologic findings, tumor differentiation, tumor size, estrogen receptor status, axillary node status, resection margins, number of pathologically examined nodes, surgical treatment, re-excision, adjuvant hormone treatment, and chemotherapy and radiation therapy. Followup information was acquired from hospital and office records, patients, and their families. The last date of followup and the date of local or distant recurrence were recorded. The local and distant disease free survival rates were then calculated from the date of definitive surgery. For estimates of local and distant disease recurrence rates, patients in whom a recurrence did not develop were censored at the last followup or death, whichever occurred first. 

Patients over 71 years of age who were undertreated by conventional criteria were compared to their appropriately treated counterparts. Our criteria for undertreatment included (1) omission of axillary sampling in patients with invasive tumors; (2) lack of postoperative radiation therapy in patients treated with breast conserving surgery; (3) lack of hormonal treatment in estrogen receptor positive patients with invasive cancers; (4) lack of chemotherapy in node-positive patients; (5) lack of chemotherapy in estrogen receptor negative patients with tumors larger than 2 cm. 

The data was analyzed using SPSS software (SPSS Inc., Chicago, IL, USA) run on a Dell personal computer. The patients were divided into two groups by age (including the age of 71 and over or younger than age 71) and compared. The significance of differences in categorica variables was evaluated using chi-square test, and the significance of differences in continuous variables was evaluated using Student's *t*-test. Cumulative 5-year local and distant disease free survival rates were calculated using Kaplan-Meier method [12]. Cox's proportional hazards regression model was used to evaluate the relative prognostic significance of variables for both local and distant disease free survival [13]. 

## 3. Results

The 2,447 patients ranged in age from 22 to 96 years and 382 (16%) were of age 71 and above, considered elderly (Table 1). The 2,065 younger patients ranged in age from 22 to 70, with a median age of 53 and the patients over 70 years ranged in age from 71 to 96 years with a median age of 76. Most patients presented with a palpable mass (37%). Patients younger and older than 71 years were equally likely to have mammographic findings. Older patients presented more frequently with mammographic masses while younger patients presented more frequently with mammographic calcifications. Both the elderly and the younger patients were most commonly diagnosed by excisional biopsy followed by core needle biopsy and fine-needle aspiration. 

Numerous significant differences were observed between the elderly and younger patients in terms of their pathology (Table 2). Older patients had significantly more infiltrating lobular cancers and fewer cases of ductal carcinoma in situ than younger patients and significantly fewer poorly differentiated cancers. The mean tumor size was significantly smaller in the elderly but the T stage distribution among the elderly and younger patients was comparable. Estrogen receptor positivity was more frequent among the elderly.

Axillary node sampling, sentinel node excision, or axillary dissection was more frequent in younger patients with removal of more lymph nodes with the proportionately more nodes involved with tumor. In addition to less aggressive treatment of the axilla, elderly patients also received less aggressive surgical treatment of the breast: only 14% received mastectomies compared to 25% of younger patients.

Adjuvant therapy with both radiation and chemotherapy was significantly less frequent in the elderly while the use of Tamoxifen or an Aromatase inhibitor was more frequent. 81% of the 1,529 young patients treated with breast conservation received radiation therapy compared to 53% of the 327 elderly patients treated with breast conservation (*P* < 0.001). Among patients with invasive cancers, 18% of the young patients received neoadjuvant chemotherapy and 33% adjuvant chemotherapy compared to 6% and 7% of the comparable elderly patients (*P* < 0.001). The main form of systematic therapy for the elderly patients was hormonal: either Tamoxifen or Aromatase inhibitor. 67% of elderly patients were treated with hormonal therapy compared with 56% of younger patients (*P* < 0.001). Despite these differences, the elderly and younger patients had similar 5-year local and distant recurrence-free survival (Table 3).

Undertreated elderly patients were identified as described in Section 2. Undertreatment consisted of omission of radiation therapy in 154 of the 317 patients treated with breast conservation, omission of axillary node sampling in 61 of the 310 elderly patients with invasive cancers, omission of chemotherapy in 10 of 63 elderly patients with involved nodes, and omission of hormonal therapy in 59 of 321 elderly patients with estrogen receptor positive cancers. By these criteria many patients were undertreated with more than one modality. As a consequence, 190 (51%) of the elderly patients were undertreated with at least one modality. Undertreated elderly patients were significantly older than their appropriately treated counterparts (77 versus 75, *P* < 0.001). The cancers of the undertreated elderly were more frequently in situ, better differentiated, smaller, and more often estrogen receptor positive (Table 4). Reflecting the criteria used to identify undertreated patients, one-third did not receive axillary sampling for invasive cancers, two-thirds did not receive radiation, almost half did not receive hormonal therapy, and a few received chemotherapy. Despite these differences in treatment, elderly undertreated patients generally fared as well as the appropriately treated elderly (Table 5). Equal numbers of patients in both groups developed local recurrences resulting in five-year cumulative local disease-free rates of 93% for the appropriately treated and 91% for the undertreated. 9% of the 167 appropriately treated elderly patients with invasive cancers developed distant disease compared to 4% of undertreated patients causing the cumulative five-year distant disease free rate to be 89% in appropriately treated patients compared to 93%   in the undertreated one. It is important to note that 44% of the 190 undertreated elderly died without disease recurrence compared to 29% of the appropriately treated patients (*P* < 0.001). 

A Cox regression model was used to evaluate potential prognostic factors such as tumor pathology, differentiation, size, number of involved nodes, estrogen receptor status, and treatment with chemotherapy, hormonal therapy, and radiation, among the elderly patients (Table 6). Local disease-free survival was significantly related to estrogen receptor status (*P* < 0.001) and pathology (*P* = 0.043). Twenty-four percent of the 46 patients with estrogen receptor negative cancers developed local recurrence within five years compared to 3% of the 275 patients with estrogen receptor positive tumors. The cumulative five-year risk of local recurrence in patients with ductal carcinoma in situ was 4% (2/66) compared to 10% (3/45) in patients with invasive lobular cancers and 9% (18/266) in patients with invasive ductal cancers. Among patients with invasive cancers, tumor size (*P* = 0.006), number of involved nodes (*P* < 0.001), and estrogen receptor status (*P* = 0.008) were significantly related to distant recurrence. Undertreatment was not significantly related to local or distant recurrence in univariate or multivariate analysis.

Undertreatment with radiation in elderly patients that underwent breast conservation was associated with increased risk of local recurrence. Five-year local disease-free survival of the unirradiated patients was 90% compared to 96% for the irradiated patients (*P* = 0.450). The cumulative five-year distant disease free survival of patients receiving chemotherapy was 73% compared to 93% for patients not receiving chemotherapy (*P* = 0.004). This difference is attributable to the larger, more poorly differentiated cancers with more positive nodes among patients receiving chemotherapy. Omission of hormonal therapy in estrogen receptor positive patients resulted in a lower distant disease free survival: 91% of estrogen receptor patients treated without hormonal therapy were without distant metastases at five years compared to 94% of patient using hormonal therapy.

## 4. Discussion

This study found that elderly patients with breast cancer present with palpable masses and mammographic findings similar to younger patients, although mammographic masses were more frequent in the elderly and mammographic calcifications were more frequent among the young patients. Cancers of the elderly tended to be less often in situ than in younger patients but invasive cancers were generally smaller, better differentiated, more frequently estrogen receptor positive, and with less nodal involvement. Older patients were treated less aggressively than younger patients. They received fewer mastectomies, less radiation after breast conservation, and very seldom did they receive chemotherapy even for node-positive cases. Elderly patients received hormonal therapy as frequently as younger patients. Despite often being undertreated, elderly patients experienced outcomes comparable to younger patients presumably because their cancers were smaller, better differentiated, and with fewer involved nodes.

More than one-half of our elderly patients were also undertreated according to current breast cancer treatment guidelines: omission of axillary sampling in patients with invasive cancers, omission of radiation in patients treated with breast conservation, omission of chemotherapy in patients with involved nodes, or omission of hormonal therapy in patients with estrogen receptor positive cancers. Despite the large number of undertreated patients, there were no significant differences in local or distant disease free survival among undertreated and appropriately treated patients. 

Previous studies of elderly patients with breast cancer have not universally observed that cancers in the elderly are biologically more favorable and less advanced than those seen in younger patients. This is in part due to differences in the populations studied. Generally when one compares the cancers of patients over 70 to patients between 50 and 70, differences are not striking [14, 15]. However, if one includes all patients younger than 70, the more favorable biology becomes more apparent [16]. In addition, many studies included elderly patients who were not treated with surgery for a variety of reasons including comorbidity, advanced disease, and patient refusal [17–20]. All of the patients in the current study were potentially curable at presentation; all had surgery, and no stage IV patients are included. A universal finding in all the studies is the increasing frequency of estrogen receptor positivity with increasing age. This usually results in the increased use of hormonal therapies in the elderly.

Undertreatment of the elderly is also a universal finding. In fact several authors have found that undertreatment, that is, lack of adherence to guidelines, is frequent at all ages [14]. The controversy that exists is whether undertreatment of patients, particularly the elderly, results in adverse outcomes. There is no question that radiation therapy reduces local recurrence rates after breast conservation for invasive and in situ disease regardless of the patient's age. However, a reduction of 3% in local recurrence does not significantly benefit an 80-year-old woman with a life expectancy of ten years who has only a 50% chance of experiencing the benefit of radiation therapy [21]. Another consideration is that patients who are not irradiated and develop local recurrences may be candidates for relumpectomy with or without radiation therapy, whereas patients who develop local recurrences after treatment with radiation should undergo mastectomy.

Previous studies noted that elderly patients with invasive cancers experience higher mortality when axillary dissection is omitted [22]. Among these studies, a few measured breast cancer specific survival. It is likely that patients not undergoing axillary dissection have higher comorbidities causing the higher mortality, not that the omission of axillary surgery caused the higher mortality. The recently completed trial randomizing patients with involved sentinel nodes to completion axillary dissection versus no additional surgery showed no benefit for completion axillary dissection [23].

Finally, with respect to chemotherapy, a few elderly patients are willing to participate in randomized trials with chemotherapy arms and a few are willing to accept chemotherapy even with relatively advanced disease [3, 4, 24, 25]. Only 36 of our elderly patients were estrogen receptor negative and 13 of these had nodal involvement. All received chemotherapy and an additional 11 patients with node negative estrogen receptor negative larger cancers received chemotherapy. Because of the small numbers of patients and the association of chemotherapy with advanced estrogen receptor negative disease, patients receiving chemotherapy fared worse than patients not receiving chemotherapy. 

This study has several limitations. It is a retrospective single surgeon database review and thus carries the inherent limitations of an observational study. This includes a potential physician bias and bias as a result of confounding by indication. It must be mentioned, however, that in today's world of cancer treatment, care is individualized and the patient ultimately determines what treatment she is to receive. A larger multicenter, prospective randomized trial of adherence to guidelines for the treatment of breast cancer in elderly patients would be needed to overcome these biases. This trial, however, is unlikely to occur and probably does not need to. Breast cancer in elderly patients has a favorable biological profile and therefore treatment does not need to fall under the confines of traditional guidelines. Moreover, coupled with comorbid conditions that are frequently encountered as people age, optimal treatment should be determined largely by clinical judgement on a case by case basis. It is known that elderly patients are undertreated but this study did not find that the omission of conventional surgery or adjuvant therapies adversely affected outcome among patients over 71 years of age. 

## Figures and Tables

**Table 1:** Comparison of demographic variables in patients <71 years and ≥71 years.

Demographic variable	<71 y	≥71 y	*P* value
*n*	2065	382	
Age (y)	53	76	
Presentation	(*n* = 2065)	(*n* = 382)	
Palpable mass	766 (37%)	137 (36%)	<0.001
Mammographic calcium	471 (23%)	62 (16%)	
Mammographic mass	434 (21%)	138 (36%)	
Mammographic abnormality	27 (1%)	1 (0.26%)	
Other	367 (18%)	44 (12%)	
Mammography: positive/suspicious	1757/1869 (94%)	326/348 (94%)	0.813
Diagnostic method	(*n* = 1933)	(*n* = 363)	
Excisional biopsy	768 (40%)	157 (43%)	0.256
Fine-needle aspiration	445 (23%)	87 (24%)	
Core needle biopsy	720 (37%)	119 (33%)	

Data are presented as *n*, median, or *n* (%).

**Table 2:** Pathologic findings in patients <71 years and ≥71 years.

Pathologic finding	<71 years	≥71 years	*P* value
Histopathology			
Infiltrating ductal	1408 (68%)	265 (69%)	0.028
Infiltrating lobular	164 (7.9%)	45 (12%)	
Ductal carcinoma in situ	424 (21%)	66 (17%)	
Unknown	69 (3.3%)	6 (1.6%)	
Tumor differentiation			
Well	319 (15%)	60 (17%)	0.000
Moderately	855 (41%)	207 (54%)	
Poorly	650 (31%)	84 (22%)	
Unknown	241 (12%)	31 (8.1%)	
Tumor size (cm)*			
Median	1.4	1.2	0.015
0–2	1428 (69%)	276 (72%)	0.250
2.1–5	398 (19%)	75 (20%)	
>5.1	117 (6%)	14 (3.7%)	
Unknown	122 (6%)	17 (4.5%)	
Node positive^†^	486/1524 (32%)	61/249 (25%)	0.027
Involved nodes^†^			
Mean	3.9	3.7	0.705
0	1137 (69%)	201 (76%)	0.066
1–3	320 (20%)	37 (14%)	
4+	180 (11%)	26 (10%)	
Estrogen receptor positive	1314/1702 (77%)	275/321 (86%)	<0.001
Initial resection margin: close/involved	751/1898 (40%)	225/353 (64%)	<0.001
Final Margin: close/involved	134/1863 (7.2%)	43/344 (13%)	0.002
Examined nodes (mean)	6.9	5.7	0.002
Axillary node sampling^†^	1524/1572 (97%)	249/310 (80%)	<0.001
Surgery			
Breast conservation	1529 (74%)	327 (86%)	<0.001
Mastectomy	519 (25%)	55 (14%)	
Unknown	17 (0.8%)	0 (0%)	
Neoadjuvant chemotherapy	280/1572 (18%)	20/310 (6.4%)	<0.001
Postoperative chemotherapy	517/1572 (33%)	23/310 (7.2)	<0.001
Tamoxifen/aromatase Inhibitor	1011/1809 (56%)	244/364 (67%)	<0.001
Tamoxifen among estrogen receptor positive patients	912/1217 (75%)	214/273 (78%)	0.231
Radiation therapy	1328/1442 (92%)	184/365 (50%)	<0.001
Radiation therapy in breast conservation	1232/1529 (81%)	173/327 (53%)	<0.001

Data are presented as *n* or *n* (%). (*Size of invasive component. ^†^Invasive tumors).

**Table 3:** Local and distant disease-free survival.

Recurrence/age	*n*	Recurrence	Cumulative 5-year recurrence-free survival (%)	HR [95% CI]	*P* value*
Local recurrence					0.563
<71 year	2065	108	93	1
				[reference]
≥71 year	382	23	92	0.95 [0.63–1.45]	
Distant recurrence					0.464
<71 year	2065	168	89	1
				[reference]
≥71 year	382	24	91	1.05 [0.75–1.48]	

*P value is from log-rank test comparing Kaplan-Meier survival curves.

**Table 4:** pathologic findings in undertreated and properly treated aged ≥71 years.

Pathologic finding	Full treatment	Undertreated	*P* value
Histopathology			
Infiltrating ductal	149 (80%)	114 (60%)	<0.001
Infiltrating lobular	18 (10%)	25 (13%)	
Ductal carcinoma in situ	15 (8%)	51 (27%)	
Unknown	4 (2.2%)	0 (0%)	
Tumor differentiation			
Well	26 (14%)	35 (18%)	<0.001
Moderately	91 (49%)	113 (59%)	
Poorly	57 (31%)	24 (13%)	
Unknown	12 (6.5%)	18 (9.5%)	
Tumor size (cm)*			
Median	1.4	1.0	0.003
0–2	128 (69%)	146 (77%)	0.169
2.1–5	41 (22%)	32 (17%)	
>5.1	8 (4.3%)	4 (2.1%)	
Unknown	9 (4.8%)	8 (4.2%)	
Involved nodes^†^			
Mean	1.1	0.5	0.021
0	119 (75%)	80 (81%)	0.103
1–3	19 (12%)	14 (14%)	
4+	21 (13%)	5 (5%)	
Estrogen receptor positive	133/162 (82%)	140/155 (90%)	0.034
Final margin: close/involved	19/166 (11%)	23/175 (13%)	
Examined nodes (mean)	8	3	<0.001
Axillary node dissections^†^	153/167 (92%)	90/139 (65%)	<0.001
Surgery			
Breast conservation	151 (81%)	167 (88%)	0.091
Mastectomy	34 (18%)	23 (12%)	
Unknown	1 (0.5%)	0 (0%)	
Postoperative chemotherapy	43/184 (23%)	5/190 (3%)	<0.001
Tamoxifen	142/181 (78%)	98/183 (54%)	<0.001
Radiation therapy	155/184 (84%)	29/89 (33%)	<0.001

Data are presented as *n* or *n* (%).

*Size of invasive component.

^†^Invasive tumors.

**Table 5:** Local and distant disease-free survival in undertreated and properly treated patients aged ≥71 years.

Recurrence/treatment	*n*	Recurrence	Cumulative 5-y recurrence-free survival (%)	HR [95% CI]	*P* value*
Local recurrence					0.847
Undertreated	190	11	91	1
				[reference]
Properly treated	185	11	93	0.79 [0.33–1.92]	
Distant recurrence (invasive cancer)					0.155
Undertreated	139	6	93	1
				[reference]
Properly treated	167	15	89	2.03 [0.86–4.80]	

*P value is from log-rank test comparing Kaplan-Meier survival curves.

**Table 6:** Cox regression of potential prognostic factors in patients over 70 (*P* values).

Variable	Local recurrence	Distant recurrence
Histopathology	0.043	0.702
Tumor differentiation	0.855	0.571
Tumor size (cm)	0.520	0.006
Involved nodes^†^	0.306	<0.001
Estrogen receptor positive	<0.001	0.008
Postoperative chemotherapy	0.517	0.198
Tamoxifen	0.091	0.375
Radiation therapy	0.620	0.375
Undertreatment	0.150	0.818

^†^Invasive tumors.
